# A Study on the Application of Distributed System Technology-Guided Machine Learning in Malware Detection

**DOI:** 10.1155/2022/4977898

**Published:** 2022-02-23

**Authors:** Shi Jin, Zhaofeng Guo, Dongli Liu, Yanhua Yang

**Affiliations:** ^1^State Grid Hubei Electric Power Co., Ltd, Wuhan 430000, China; ^2^Grid Hubei Electric Power Co.,Ltd, Wuhan 430000, China; ^3^College of Engineering, Fujian Jiangxia University, Fuzhou 350108, China

## Abstract

In recent years, with the development of information technology, the Internet has become an essential tool for human daily life. However, as the popularity and scale of the Internet continue to expand, malware has also emerged as an increasingly widespread trend, and its development has brought many negative impacts to the society. As the number of types of malware is getting enormous, the attacks are constantly updated, and at the same time, the spread is very fast, causing more and more damage to the network, the requirements and standards for malware detection are constantly rising. How to effectively detect malware is a research trend; in order to tackle the new needs and problems arising from the development of malware, this paper proposes to guide machine learning algorithms to implement malware detection in a distributed environment: firstly, each detection node in the distributed network performs anomaly detection on the captured software information and data, then performs feature analysis to discover unknown malware and obtain its samples, updates the new malware features to all feature detection nodes in the whole distributed network, and trains the random forest-based machine learning algorithm for malware classification and detection, thus completing the global response processing capability for malware. By building a distributed system framework, the global capture capability of malware detection is enhanced to robustly respond to the increasing and rapid spread of malware, and machine learning algorithms are integrated into it to achieve effective detection of malware. Extended experiments on the Ember 2017 and Ember 2018 databases show that our proposed approach achieves advanced performance and effectively addresses the problem of malware detection.

## 1. Introduction

The rapid development and expansion of the Internet has brought huge and significant changes to the whole society. At the same time, malware is also becoming more prevalent and increasingly damaging to the network. Malware is a major security risk in the field of computers and networks and a research topic focused on by information technology researchers. Users' private data, personal information, and property are all targets of malware attacks, which can cause serious damage to computer and network systems. Malware can propagate quickly throughout the network and is not easily detected, and its spread is fast and causes great harm [1]. As people have become more knowledgeable about computers and networks, it has become easier and easier to prepare more sophisticated malware. Its speed and destructive power can make the Internet's backbone network seriously blocked or even paralyzed.

**Figure 1:**
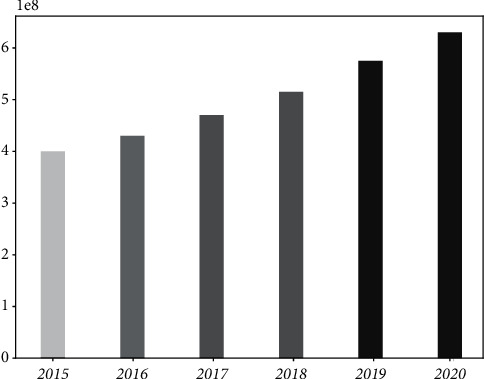
Malware growth trend chart. Malware on the Internet shows a rapid increase in unfavorable conditions every year.

The increasing number and variety of malware on the network are very damaging to the network. As shown in Figure 1, malware on the Internet shows an unfavorable situation of rapid growth every year. The emergence of new malware is always sudden and spreads very rapidly, making malware detection under large-scale networks very difficult. However, the main path of malware propagation is still the network, so a distributed architecture can be used to detect key network nodes and collaborate among nodes to achieve detection, control, and prevention of malware in a large-scale network environment [2]. The classification mining system of big data is a multistep collaborative system, with both local analysis and global pattern discovery at the node level, and the focus of the problem is different at different stages. For example, the local node processing should emphasize real-time and efficient processing in the face of potential fast-moving malware attacks that accumulate over time. In contrast, the most important task of the global detection model is to construct classifiers that can be shared globally, so more emphasis should be placed on the predictive capability and interference resistance of the model and so on. Therefore, the corresponding theories and methods should be studied according to the needs of different malware stages to form a solution with integrated technology and systematic methods. Distributed malware detection has received widespread attention, in which fundamental research has become an active branch in malware detection. Distributed malware detection results in the most single data stream detection methods, of which incremental mining has been widely paid attention to as an effective method for dynamically mining streaming data that flows over time.

Traditional malware detection techniques often require a lot of manual involvement, such as signature-based detection techniques and heuristic detection techniques, which require many detection rules to be developed manually. Such traditional detection techniques are not good at detecting new types of malware that have not been seen before. This is especially true when the number of malware additions is high on a daily basis. In recent years, with the boom in machine learning, malware detection techniques have begun to be combined with machine learning, which we call malware intelligent detection techniques. This new detection technology can better detect new and previously unseen samples and better meet the security needs of today's society. As a representative of the new malware detection technology, malware intelligent detection technology has the feature of strong generalization ability and also can reduce human involvement to a certain extent [3]. Therefore, the study of effective malware intelligent detection technology has a high value. For malware intelligent detection-related research, how to effectively apply machine learning technology is one of the keys. From a macropoint of view, the application of machine learning techniques needs to include 3 main steps: feature extraction, feature processing, and classifier design. These 3 main steps are also the main research points of the current work related to malware intelligent detection. In the face of malware intelligent detection scenarios, related work needs to combine the characteristics of malware, targeted design of these three main links. At the same time, since it is an intelligent detection, it means that some problems faced by machine learning techniques must also be fully considered, such as collecting data sets, avoiding overfitting, and improving training speed. In this paper, we focus on how to discover known malware propagated through the network in a distributed network environment; discover unknown malware in time and analyze it centrally using machine learning algorithms; and collect and count the discovered malware, generate events and reports, and build a hierarchical model of the malware prevention and control system. According to research, static detection methods using machine learning techniques achieved an accuracy rate of over the 90%, dynamic detection methods were able to achieve an accuracy rate of over 96%, and the performance of such methods has been further improved through continued development in recent years [4]. Establishing an intelligent detection model based on machine learning technology to form a line of defense for blocking malware is a new direction for technological breakthroughs and market expansion and has important research significance and application value.

The detection of malware is completely possible using a cloud distributed parallel environment for distributed parallel detection of malware. The maximum independent set algorithm is used to optimize the network structure, avoid network congestion, and balance the load. Distributed parallel detection of suspicious software through the nodes of the distributed network improves the efficiency of detection and finally forms an analysis report that is sent to the client. This paper summarizes the malware detection methods based on machine learning and proposes a framework for malware detection by machine learning guided by distributed system technology to address the problems of slow response and lack of global analysis due to the existing malware detection methods that are difficult to cope with the large-scale network growth. The main contributions are as follows: (1) A new framework for distributed malware detection in a large-scale network environment is proposed. The newly emerged malware is analyzed, and its features are extracted and then updated to all nodes of the system, thus quickly achieving the detection of this malware in the whole system jurisdiction. (2) A new machine learning malware detection method is proposed to reduce the dimensional of the malware feature vector using the PCA algorithm. The random forest algorithm is further designed to obtain detection results by classifying malware using its robustness and strong fitting ability. (3) Experiments are conducted on Ember 2017 and Ember 2018 datasets, and the outstanding performance of the proposed method and its detection capability in large-scale networks are verified by comparing and analyzing the experimental results. Finally, a conclusion and future perspectives are provided.

## 2. Related Work

### 2.1. Distributed Architecture

Earlier detection of computer malware was done on the host computer in a completely isolated and controlled environment that did not require collaboration. However, network malware spreads throughout the Internet, an environment that cannot be isolated and is not fully controllable. Especially today, with the rapid growth of the Internet and the increasing size of networks, it is impractical to isolate each network in turn and then remove the malware. Collaboration can also include analysis of anomalies, notification of response methods, and feature broadcasting. Qiu et al. [5] gave the main problems and countermeasures to be faced by distributed mining of data streams, which is one of the earlier and more comprehensive pieces of literature on distributed mining techniques for data streams. One of their important points is that effective use of limited computer processing resources to solve the knowledge discovery problem of potentially infinite data requires finding a distributed solution that balances cost and accuracy, while Aslan and Samet [6] illustrated that distributed mining of data streams requires a comprehensive consideration of distributed computing, memory buffering, and node interaction costs from the perspective of performance optimization. They also designed a hierarchical mining architecture for data streams; that is, the mining system consists of several local nodes and one central node, the primary patterns are formed by parallel mining of local nodes, and the central node then generates global knowledge patterns. These studies, especially the mining idea of cost-accuracy balance, are also the key issues to be considered when designing the mining model in this paper. Of course, the distributed mining of data streams is concerned mainly with how to use distributed parallelism to solve the knowledge discovery problem of large-capacity (single) data streams. In fact, with the popularity and growth of network-based computer applications, multiple data streams with multiple nodes independently clustered but logically related have become another important data form, that is, distributed data streams. Admittedly, most of the current literature on distributed data stream mining is still focused on the study of key scientific problems and solutions, but as the scientific problems become clearer, some related mining architectures and methods have been discussed in recent years. Alazab et al. [7, 8] proposed a mining algorithm DS-means for distributed data streams. The typical unsupervised learning algorithm K-means is used in both local and global clustering of DS-means. However, in order to accommodate the capacity fluctuations of different data streams on different nodes and different time periods, the number of global class clusters is dynamically adjusted based on the clustering results of local nodes before global node clustering. Liu et al. [9] constructed a distributed multinode mining architecture for the problem of mining frequent itemsets for distributed data streams and designed corresponding algorithms to implement the key operations of the architecture. The work in this paper draws on these distributed data stream mining architectures and methods. In short, distributed data stream mining is a natural extension and development of the research on data mining and its data stream mining techniques, and the current research is mainly focused on algorithm design. However, in the face of the growing demand for big data, systematic research on the mining architecture of big data and its core mining models and algorithms under the related architecture becomes a problem that must be faced. In this sense, distributed data streams can provide an ideal model of data organization for big data in many application environments, and mining algorithms for distributed data streams can be used as one of the core techniques to address big data mining.

In fact, as the concept of big data is getting hotter and hotter, some scholars or research institutions have started to explore the knowledge mining problem in big data experimentally. Gupta and Rani [10] argued that the mining of big data needs to study different algorithms at different processing stages and designed corresponding algorithms for some key steps. Naeem et al. [11] discussed the problem of big data analysis from the data mining viewpoint and explored the problem of big data based on data-driven strategies. And based on the data-driven strategy, they explored the core problems in big data mining and designed an architecture and core model for big data mining. Pan et al. [12] argued that big data has two data forms, batch and streaming processing, and analyzed the key technologies in big data streaming computing in a more systematic way. Big data mining is also a balanced optimization problem of cost and accuracy, where improving the accuracy of distributed mining with reasonable communication cost is one of the key scientific problems. Euh et al. [13] proposed an idea of mining microcluster patterns in data streams, that is, extracting statistical values such as points and means of each cluster after performing clustering algorithms on a data stream to form the so-called microcluster patterns. Amer and Zelinka [14] designed the statistical information of microcluster-like data into a tree structure that grows with time to maintain it. Both of these works are oriented to single data stream mining. A related approach directly oriented to distributed data stream mining comes from the work of Omer et al. [15]. Although microcluster mining is effective at local nodes, microcluster patterns are not suitable as global patterns because they lack sufficient predictive power, that is, the ability to classify unknown data. In fact, in distributed data stream classification mining, although data are collected and decentralized at local nodes, the data streams scattered at local nodes are interconnected, so discovering global classifiers for multinode sharing and prediction is one of the main tasks of classification mining for distributed, streaming big data. In addition, since distributed mining has to go through local pattern mining and data transmission before global pattern mining, the multistep processing may lead to degradation of data quality, so the selection and design of global patterns must also consider the noise immunity performance. Based on the above two points, considering the high predictive capability and better robust performance of the integrated learning technique, this paper will study the malware detection methods suitable for the distributed approach by drawing on the existing integrated learning technique.

### 2.2. Based on Machine Learning Methods

Early detection and analysis of malware were performed by security experts using various tools to assist in tracking, tuning with disassembly to obtain the behavior of the software, and then combining their experience to give analysis results. This manual analysis method is technically mature, and if security personnel are experienced, the possibility of error is very small. But the analysis efficiency is very low and has been unable to meet the rapidly growing needs of malware analysis, so security vendors and researchers have proposed a variety of automatic detection and analysis methods. Now the research on malware is divided into four main categories: (1) malware detection, the study of the real function and purpose of software execution; (2) analysis, according to the detection results of the detection object automatically classified; (3) prevention, to prevent malware running in the computer; (4) recovery, if the malware has invaded the system, remove it from the system and repair the damaged system; the ideal antimalware system should include all of the above functions. However, the current security software for ordinary users only has the functions of categories (1) and (3), and only professional security software has the functions of category (4), while the software of category (2) is still in the research stage. According to the different perspectives, malware detection methods are divided into two types of static detection and dynamic detection; the following are the two types of methods, the existence of the differences and their respective advantages and disadvantages.

Static detection is a method that gives analysis results based on the characteristics of the program being tracked and analyzed without being run. The earliest use of static detection technology is currently used by ordinary users of various types of antivirus software, which has a history of 30 years. It is established manually by the security vendor to create a malware feature library, the antivirus software only needs to compare the software being detected with the software in the malware feature library, and you can conclude whether it is malware. The sent kind of eigenvalue scanning technology is very mature, and the false positive rate is very low. However, the establishment of a malware repository requires security vendors to invest a lot of manpower and resources and keep it updated in real time. And with more and more malware, the malware library is becoming more and more huge and large, the scanning and analysis speed is getting slower and slower; more seriously, this technology can only identify malware captured by the vendor and the timeliness is relatively poor, so it is no longer the focus of industry research. Currently, widely studied static detection technology is the use of machine learning algorithms for detection and classification.

Cai et al. [16] first introduced Bloodhound technology, which uses heuristic algorithms to analyze software, with a certain degree of machine learning, can use existing knowledge of the software, and has not been manually analyzed to give analysis results; then the eigenvalue scanning method is more time-sensitive, no longer completely dependent on malware libraries. Kouliaridis et al. [17] proposed a standard compressed distance method to measure the similarity between malware and then classify them. Hei et al. [18] used the system API call sequence as the basis for determining malware, which can be able to restore the deformed call software dynamically, partially solving the software deformation problem. Liu et al. [19] used the program data stored in the PE format file to analyze and obtain the DLL file from which the program calls the system. Huang et al. [20] proposed an inference tree-based analysis method. They used the *k*-testable tree to differentiate the data flow of software to obtain a relationship graph between the data and use this as a basis for classifying malware. Liu et al. [21] studied the use of variable-length *n*-grams as classification features, and the experimental results showed that variable-length *n*-grams as classification features can achieve higher accuracy. Ozsoy et al. [22] studied text classification techniques to improve the traditional feature code scanning method and proposed an automatic *n*-gram-based detection method, which has leading experimental results. Galal et al. [23] conducted an experimental comparison of malware analysis techniques used to test a variety of mainstream classifiers, including KNN and NB. Naval et al. [24] proposed an attribute-based automatic detection method. It first statically extracts the *n*-gram information of the program and then uses the information gain value as the criterion for feature selection; then, the extracted features are reduced to eliminate redundant features, and the optimized feature vector is fed into the classifier to achieve automatic detection of malware. The static detection method based on an integrated neural network is also proposed, and the feature selection technique is introduced in the construction of the neural network to combine the perturbed input attributes and the chemistry training data to generate the neural network. The conclusion obtained is that the integrated classifier is better than the single classifier. Although scholars at home and abroad have made unremitting efforts in the field of static detection and achieved many results, static detection methods have never achieved practical results. In addition to the design of classification algorithms, there is another difficulty that can never be solved: the data used for classification cannot be guaranteed to be real.

Dynamic detection and analysis are a method of letting the program under test run and then extracting features for detection and analysis. The features here mainly refer to the behavior of the program or the result of the behavior, so it is also called behavioral feature detection and analysis. The study of dynamic detection and analysis is actually also divided into two parts: one is the acquisition of behavior (i.e., detection), and the other is the analysis of behavior. The acquisition of behavior is also divided into two types of technical solutions. Another type of real-time monitoring method is the smart Hook technology inserted between the program and the operating system or hardware device, which can directly monitor all the persistent behavior of the program, which is also the mainstream monitoring method. Using Hook technology, the industry has developed a number of types of widely used real-time behavior monitoring software; they have different functions, and each has its own strengths and becomes an indispensable helper for researchers to study the behavior of viruses. Kouliaridis et al. [17] proposed a new method of scanning memory areas using hardware. Huang et al. [20] proposed a technique to check malware based on component integrity, whose kernal also uses digital signature techniques and downloaded software components from the web. Das et al. [25] proposed a method to monitor malware using hardware virtualization and software virtualization techniques. Idrees et al. [26] proposed a new approach to protect against malware injection by using hardware secure return address heap-finding techniques to achieve this dedicated hardware heap-finding to protect legitimate programs at the hardware level at a minimal cost to the system, complementing traditional software buffer overflow prevention methods. To analyze the kernel program, Omer et al. [15] proposed to compare whether the system kernel is modified by malware by taking a snapshot of the kernel environment. This method is not easily detectable by malware. To detect malware, Euh et al. [13] proposed a technique called confusion call detection. The algorithm was improved by Idrees et al. [26] by using a “sliding window” to improve the efficiency of the algorithm. The basic idea is to measure the similarity between sequences by calculating edit distance and then use the KNN algorithm to cluster the malware. Demontis et al. [27] used the bag-of-words model to parse program behavior, automatically extract the feature words, and convert these feature words into vector space data and then used support vector machines to achieve the automatic classification of malware. Yewale and Singh [28] proposed the use of an expert system for malicious behavior determination, which evaluates all behaviors as a whole, considers their potential maliciousness, and calculates the overall result. However, they only proposed such an idea and did not give a concrete implementation of it. Based on these studies, experimental systems have been developed in several research institutions. In general, although the current program behavior-based pickup and analysis method is not as commonly applied as the static analysis method, it has some advantages that the latter does not have, so the development prospect is more promising, and it is also the main direction of researchers at present.

## 3. Method

**Figure 2:**
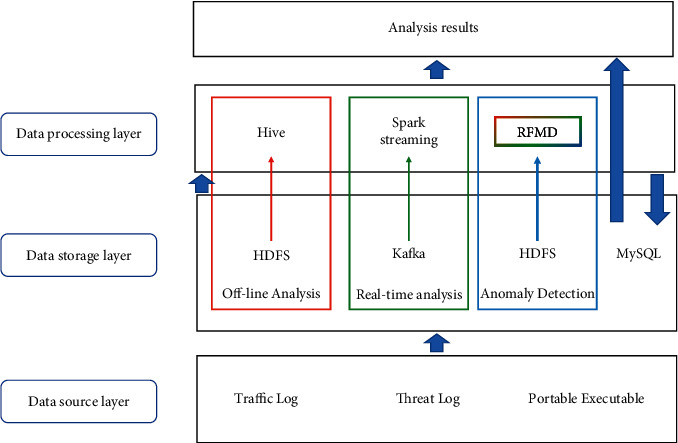
Distributed system technology-guided machine learning in malware detection architecture.

The overall structure of our proposed approach is shown in Figure 2. In Section 3.1, we first describe the architecture, components, and main functions of the proposed distributed system. For the subnodes in the distributed system, we describe in detail their algorithms for performing feature extraction and random forest-based malware detection. In Section 3.2, we describe in detail the proposed random forest-based malware detection method.

### 3.1. Distributed Architecture

The system uses a hierarchical model of distributed detection and collaborative analysis management. In the system, detection nodes are arranged on the nodes of each large-scale network, and the probes of the nodes are placed on the network entrances and exits to be monitored. The node module consists of a feature detection module, anomaly detection module, and analysis control module. The feature detection module detects various malware with known characteristics, and the anomaly detection module is responsible for finding anomalies. They are connected through the analysis control module, while each node communicates and collaborates with each other through the analysis control module, thus framing a distributed and collaborative malware detection system on a large-scale network. The following describes the model for constructing a distributed malware detection system based on node detection.

#### 3.1.1. Node Detection

At the access point of the network, the data is bypassed into the processing system through the port mapping of hub or switch. At each node, the analysis function of the node is divided into two parts, and the data captured by the probe is sent to both anomaly detection and feature detection modules: (1) processing of known malware, using the existing feature library to detect known malware in the network; (2) analysis of the data using the anomaly discovery module, from which malware not yet in the library is found. The results of the analysis and the data to be further analyzed are sent to the analysis console. As the functions of the two modules differ greatly, the detection of known malware is relatively fixed, while the detection of the unknown can be selectively added or removed by means of anomaly discovery according to the actual situation. The detection points can be dynamically added and reduced according to the actual situation when constructing the network topology model without changing the network settings or increasing the load on the network. This division makes the system composition flexible and extensibility.

#### 3.1.2. Distributed Topology

**Figure 3:**
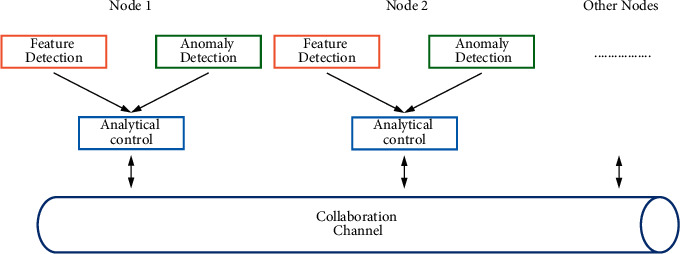
Sketch map of distributed cooperation.

Due to the system's distributed detection approach, the system is highly extensible, and the architecture is extremely flexible, allowing the system to scale effortlessly when monitoring across networks, thus enabling distributed interoperability, which is of great importance for Internet-wide malware detection. The distributed collaboration of the system is shown in Figure 3.

In Figure 3, each node is able to perform the detection task independently; at the same time, it can communicate with other nodes through the collaboration channel. When a new malware appears in a subnet, it is captured by the detection nodes in the subnet and quickly analyzes the characteristics; then, it is notified to other nodes through the collaboration channel so that other nodes have the ability to detect such malware. The connection relationship between analysis and control modules can be a simple peer-to-peer relationship, or a certain loosely coupled hierarchical relationship can be structured, which does not affect the working of the system. High-level analysis control modules are simply application-based definitions that function and implement exactly the same as other analysis control modules. The analysis results of multiple single nodes can be reported to the analysis control platform; the analysis console can synthesize the situation of each node for analysis, thus giving a global view of the analysis. In turn, the regional analysis console can send the data that needs to be analyzed to the advanced analysis console so as to obtain powerful analysis support and improve the system analysis performance when the analysis task cannot be completed. In each node of the backbone network, a packet-catching probe and malware analysis platform are placed to analyze the captured data, from which anomalies are discovered and the results are sent to the analysis control center in the region. Each regional node is connected to a regional analysis control center through a dedicated line; within the entire controlled network, all analysis platforms are connected to an advanced analysis control center.

#### 3.1.3. System Functions

**Figure 4:**
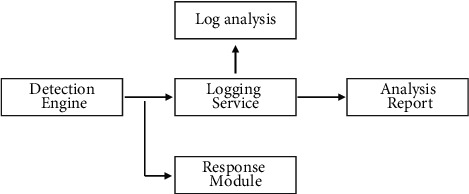
Signature detection of malware.

The functional components of the malware signature detection module and anomaly detection module, as well as the analysis and control module, are described as follows:

1. Malware feature detection module: As there are many various known malware currently prevalent on the network, their behavioral characteristics are well known, and their signature codes have been obtained; their detection is performed through known modules, such as CIH. The block diagram of the known detection module is composed as shown in Figure 4. The data stream enters the detection engine, which analyzes the data and writes logs if known malware is found and notifies the response module to perform analysis of the logs to generate reports for publication on the Web. The functions of the modules are as follows.
2. Detection engine: According to the content of the feature database, analyze the data obtained by the capture platform; if a match with known features is found in the data, notify the response module and carry out the corresponding response action; at the same time, record the generated logs. Log information includes source IP, destination IP, source port, destination port, and type of malicious code. Log service: accept the analysis results generated by the engine, write the results into the database, and publish them through the Web. Log analysis: analyze the log database, generate reports and statistics based on the data, and provide queries.
3. Statistical methods are the simplest and most feasible way to detect anomalies. Specific statistical analysis methods are currently a hot research topic and are under rapid development. Statistical analysis methods first require the creation of a statistical description that summarizes a number of measured attributes (such as source address, destination address, and destination port counts) and records the values of these attributes during normal use. The width of the methodological confidence interval for the statistics depends on the user setting different confidence levels and does not require previous knowledge about anomalous activity. Statistical anomalies are found from the observed values, and the confidence intervals change themselves as more data are measured. Statistical analysis is based on the usual statistics, setting confidence intervals for the transmission on the network; if the measured attributes are not within the confidence interval, anomalous behavior is considered to have occurred. The advantage of statistics is that the algorithm and implementation are very simple and can detect unknown and more complex intrusions; the disadvantage is that there are false positives, high leakage rates, and low sensitivity.
4. Sampling: Real-time detection of anomalous behavior for large-scale backbone networks is constrained by system performance, mainly (1) processing speed, including the speed of network adapter hardware, protocol stack reduction, and detection algorithms; (2) storage capacity, because the backbone network bandwidth is very high and the data per second is in GB if stored. In this case, the sampling detection method can be used. This detection does not require the use of traditional methods to measure and record all traffic data. Using sampled message measurements is sufficient for real-time needs rather than measuring all traffic information. Sampling can be done at regular intervals or according to certain sampling methods. Sampling methods include random sampling and Poisson sampling. In this paper, we adopt distributed sampling and fixed-time source sampling. The sampling method is used to obtain anomalies in network traffic and network behavior. The data obtained by sampling is used for statistical analysis, for both anomaly discovery and more complex content analysis. The sampling parameters are modified so that the samples obtained by sampling describe the statistical properties of all traffic as well as possible. The advantage of sampling is that the samples can be processed and analyzed in a more focused and detailed manner; the disadvantage is that some anomalous information is easily missed.

#### 3.1.4. Analysis Control Module

**Figure 5:**
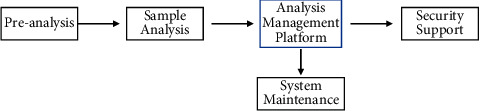
Model of anomaly detection.

The analysis control module needs to preanalyze the suspected data captured by the anomaly detection module in order to be able to discover the unknown malware in time, and if it is confirmed as malware, it has to perform sample analysis as soon as possible. The analysis object of analysis control is the suspicious data stream obtained from the anomaly detection module, and it is controlled by the next level of analysis control center, known detection module, logging service, and information distribution. The analysis control center centralizes all the control of the system, while all human operations are directly facing the analysis control center, and the analysis control center includes the work of all people. The work of each module of the analysis control center includes the following (see Figure 5).

The sequential treatment process is as follows: (1) preanalyzing, which preanalyze the suspected data; (2) sample analysis to obtain its characteristic codes and activity characteristics; (3) analysis management platform, to conduct comprehensive analysis and evaluation of the samples; (4) system maintenance, the feature code obtained by analyzing the unknown anomaly is added to the feature library of each known detection module; (5) security support, including issuing notifications to users, generating reports, and releasing special killing tools. The features of new malware acquired by the analysis control platform through the analysis of anomalies can be added to the feature base of known detection modules through the updated model of the feature base, thus enabling such malware to be detected as known. As the requirements for emergency response are getting higher and higher and the analysis and control capabilities are not balanced everywhere, it requires a coordination problem that can coordinate all human and material resources to focus on quick problem solving and emergency response, and then it can effectively reduce losses.

#### 3.1.5. Mechanisms for Collaboration

**Figure 6:**
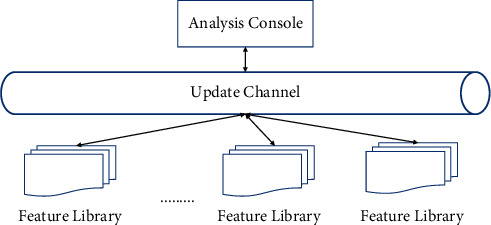
Extensibility model of signature database.

In order to be able to collaborate among the nodes and to synthesize the analysis for easy management, communication between the nodes in the system is required. The communication includes (1) analysis of unknown malware, source data, and analysis results, (2) control information, and (3) notification of analysis results and new response handling to each node. The most important feature library update is the communication between the nodes. As new malware appears faster and faster, the size of the feature library can expand dramatically and needs to be updated very timely. The system uses a centralized control structure that includes the console and its connected feature libraries for several known detection modules, as shown in Figure 6.

The console is responsible for updating all the feature bases, and two mechanisms of push and pull can be used when operating on the feature bases as follows: one update operation on the feature bases initiated by the console; the virus engine actively connects to the console to update the feature bases. When the analysis console analyzes the features of the newly discovered malware, it takes the initiative to update the feature base of the detection engine of each node, that is, push. Since new malware is not discovered in a short interval, pushing is not performed frequently, but to keep the feature base fresh, the higher-level analysis console is connected every longer period to obtain the malware features that need to be updated and remove the outdated feature codes. In this paper, we use a hierarchical and connected organization model. The analysis control center at the lower level communicates with and receives control from the higher level. At the same time, each analysis control center is autonomous, that is, fully qualified to work despite other failures.

### 3.2. Detection Algorithm

In this paper, we propose a random forest-based malware detection algorithm (RFMD). By introducing machine learning techniques, RFMD is able to accurately detect new malware and its variants. The core modules include distributed feature extraction module, feature selection and transformation module, and Spark-based random forest classification module: (1) distributed feature extraction module: based on Spark parallel computing framework, this module performs batch analysis of PE files to obtain 56 features and form a 56-dimensional feature vector; (2) feature selection and transformation module: based on PCA algorithm to process the 56-dimensional feature vector and reduce the dimensional; (3) Spark-based random forest classification algorithm.

The file header information can also be used as program characteristics, but it is usually in portable executable (PE) format. The PE file header contains numerous information about the program under test, including the number of sections, section names, section sizes, timestamps, dynamic link library information, import table information, and export table information. This information can be regarded as a macrodescription of the program to be tested. In this regard, the file header information is often used as an important feature for intelligent detection of malware. The core detection principle of the method proposed in this paper is that the feature identifiers contained in malware binary PE files can be used for automatic identification and classification of malware. The principle assumptions are described as follows: (1) In order to bypass the signature detection of AV, malware eliminates or circumvents the signature by variants, but the features contained in malware PE files are stable to a certain extent. (2) Malware causes its PE file features and structure differ significantly from benign software originating from commercial development environments. But this difference is only in functionality and does not affect its execution capability. Moreover, this difference can be used to distinguish malware from benign software to a certain extent. (3) Usually, malware uses shelling, encryption, and other obfuscation methods to bypass AV detection. (4) For rapid development as well as rapid mutation, low-level programming techniques usually trigger anomalies in PE file structure. (5) To implement functions commonly used by malware, some key combinations of API functions are usually information such as comments left in the software by malware authors, which are usually text strings. Based on the above six assumptions, the research and validation work in this paper consists of the following four steps, namely, potential effective feature design and extraction, feature simplification, feature contribution evaluation, and system evaluation. In order to enable the features in this paper to cover different segments of malware, features from different segments are extracted and subfolded, described as follows: information about the external structure of the file from the PE file header (DOS header and PE header) and information about the segments coming from inside the file structure are extracted. In summary, 56 potential features are extracted from the above description. Feature utility assessment, based on a training dataset, is performed in this paper to evaluate the ability of the 56 features in classifying malware and benign software samples, that is, classification contribution. The process results in a statistical model that describes the contribution of each feature to the classification and ultimately generates the metadata of the malware PE file. System evaluation: to validate the effectiveness of the metadata of malware PE files for malware classification, based on the Spark distributed environment, this paper implements a prototype system, RFMD, to classify a collection of test PE files, resulting in malware and benign software based on 2 test datasets.

A random forest is a classifier consisting of multiple decision trees {*h*(*x*, *θ*_*k*_)}, where {*θ*_*k*_} are mutually independent and identically distributed random vectors. The final class label of the input vector is determined by the combination of all decision trees. To construct *k* trees, we have to generate *k* random vectors *θ*_1_, *θ*_2_,…, *θ*_*k*_, which are mutually independent and identically distributed. The random vectors *θ*_*i*_ can be constructed as a decision classification tree *h*(*X*, *θ*_*i*_), which is simplified to *h*(*X*). Given *k* classifiers *h*_1_(*x*), *h*_2_(*x*),…, *h*_*k*_(*x*) and random vectors *x* and *y*, define the edge function:

(1) $$\begin{array}{c} mg\left(x,y\right)=av_{k}I\left(h_{k}\left(x\right)=y\right)-\underset{j\neq y}{\max}av_{k}I\left(h_{k}\left(x\right)=y\right), \end{array}$$

where *I*(∘) is the demonstrative function. This marginal function portrays the extent to which the average number of votes for the correct classification of vectors *x* and *y* exceeds the average number of votes for any other class. The larger the margin, the higher the confidence level of the classification. Thus, the generalization error of the classifier is as follows:

(2) $$\begin{array}{c} {\text{PE}}^{*}=P_{x,y}\left(mg\left(x,y\right)<0\right), \end{array}$$

where the subscripts *x* and *y* represent that the error is in the *X* and *Y* spaces. Extending the above conclusion to a random forest, *h*_*k*_(*X*)=*h*(*X*, *θ*_*k*_). If the number of trees in the forest is large, the following theorem can be obtained using the law of large numbers and the structure of the trees. As the number of trees increases, for all random vectors *θ*_1_, *θ*_2_,…, *θ*_*k*_, PE^*∗*^ tends to

(3) $$\begin{array}{c} P_{x,y}\left(p_{\theta }\left(h\left(x,\theta \right)=y\right)-\underset{j\neq Y}{\max p_{\theta }\left(h\left(x,\theta \right)=j\right)<0}\right). \end{array}$$

It shows that the random forest does not overfit, which is an important feature of the random forest, and the generalization error PE^*∗*^ will tend to an upper bound as the number of trees increases, which indicates that the random forest scales well to unknown instances.

Strength of classifier *h*(*X*, *θ*_*i*_) is as follows:

(4) $$\begin{array}{c} s\leq E_{x,y}mr\left(x,y\right). \end{array}$$

Assuming that *s* ≥ 0, according to Chebyshev inequality, from equations (3) and (4), we can obtain

(5) $$\begin{array}{c} {\text{PE}}^{*}\leq \frac{\mathrm{var}\left(mr\right)}{s^{2}}. \end{array}$$

The upper bound on the generalization error of the random forest is derived and defined as

(6) $$\begin{array}{c} {\text{PE}}^{*}\leq \frac{\rho \left(1-s^{2}\right)}{s^{2}}, \end{array}$$

where *ρ* is the mean value of the correlation coefficient and *s* is the classification strength of the tree. From Theorem 2, the upper bound on the generalization error of the random forest can be derived based on two parameters: the classification accuracy of each decision tree in the forest, that is, the tree strength *s*, and the degree of interdependence between these trees *ρ*. When the correlation degree *ρ* of each classifier in the random forest increases, the upper bound on the generalization error PE^*∗*^ increases; when the classification strength of each classifier increases, the upper bound on the generalization error PE^*∗*^ increases. A correct understanding of the interplay between these two is the basis for our understanding of how random forests work.

The random forest works very differently from other classifiers because the model is trained with a set of random vectors *θ*_1_, *θ*_2_,…, *θ*_*k*_. The design of this set of random vectors is to maximize the discretionary of the random forest to reduce the generalization error and improve its generalization ability. In the classification stage, the class labels are obtained by combining the classification results of all decision trees. The most currently used methods are voting and probabilistic averaging. For the test sample *x*, the predicted class label *c*_*p*_ can be obtained:

(7) $$\begin{array}{c} c_{p}=\arg\underset{c}{\max}\left(\frac{1}{N}\sum_{i=1}^{N}I\left(\frac{n_{h_{i},c}}{n_{h_{i}}}\right)\right), \end{array}$$

(8) $$\begin{array}{c} c_{p}=\arg\underset{c}{\max}\left(\frac{1}{N}\sum_{i=1}^{N}\left(w_{i}\frac{n_{h_{i},c}}{n_{h_{i}}}\right)\right). \end{array}$$

Equation (7) is the result of voting, and equation (8) is the result of probability averaging. *N* is the number of decision trees in the forest, and *I*(*∗*) is the schematic function. *n*_*h*_*i*_,*c*_ is the classification result of tree *h*_*i*_ for class *C*, *n*_*h*_*i*__ is the number of leaf nodes of tree *h*_*i*_, and *w*_*i*_ is the weight of *I* decision trees in the forest. In this paper, we use voting to determine the class labels; that is, the test set goes over one side of each tree in the forest, the detection results of each tree for each software are recorded, and the final class labels of the software are those classes that receive more votes than some threshold.

## 4. Experimentation and Evaluation

### 4.1. Datasets

To effectively validate the proposed approach, we experimented with the publicly released Ember datasets from the security company Endgame, hoping for the application of distributed systems and machine learning techniques in malware detection. The data includes include two parts, including Ember 2017 and Ember 2018, and Table 1 shows the number of samples and categories of the datasets. According to the dataset's presentation, the collected malware samples were flagged as malicious by multiple detection engines, while benign samples were not flagged as malicious up to the time of collection, and this section uses these datasets as experimental data. An additional 7994 malicious samples and 7158 benign samples were collected during 2019 to validate and compare several major approaches.

### 4.2. Experimental Setup and Evaluation Metrics

Experiments were conducted using the Python language and Spark distributed system, and the relevant environment configurations and software versions are listed in Table 2.

To evaluate the detection accuracy of the system, the performance of the model is evaluated using Accuracy, Precision, Recall, and *F*1-value (*F*1_score). Precision denotes the proportion of the data judged as malicious code that is truly malicious, defined as Precision = TP/(TP + FP). Accuracy denotes the overall precision of the detection system, defined as

(9) $$\begin{array}{c} \text{Accuracy}=\frac{\text{TP}+\text{TN}}{\text{TP}+\text{TP}+\text{FP}+\text{FN}}*100\%. \end{array}$$

Recall, which indicates the ratio of the amount of relevant information retrieved from the database to the total amount, is defined as Recall = TP/(TP + FN). *F*1-score, which is used to synthetically evaluate Precision and Recall, is the weighted summed average of Precision (*P*) and Recall (*R*). It is defined as *F*1=(2*PR*/*P*+*R*).

### 4.3. Detection Performance Comparison

We have selected competitive approaches in machine learning-based malware detection for comparison. We conducted an analysis to validate several commonly used machine learning methods, such as the proposed RFMD, LightGBM, SVM, and *K*-means.

**Figure 7:**
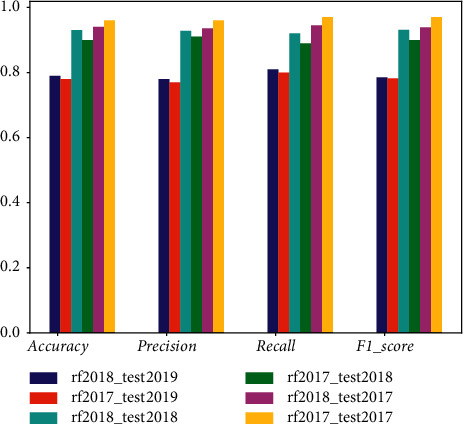
Evaluation metrics for RFMD models.

The models were cross-checked on the Ember 2017 test set, the Ember 2018 test set, and the 2019 sample set.  Figure 7 shows the results of the performance evaluation, calculating Accuracy, Precision, Recall, and *F*1_score, respectively. Based on the overall comparison, the following conclusions are drawn: (1) All models outperform their performance on the Ember 2017 test set than their performance on the Ember 2018 test set. According to the publisher's (Endgame) statement, the detection difficulty was purposefully increased when collecting the 2018 malware samples, thus producing this comparison in this experiment and reflecting that machine learning-based detection methods are still affected by the detection difficulty of the malware itself. (2) Comparing Accuracy, Precision, Recall, and *F*1_score, we can see that “rf2017” outperforms “lgbm2018” on the Ember 2017 test set; on the Ember 2018 test set, the result is the opposite. This result indicates that the coverage of the training data has an impact on the model performance, and the detection ability of the model relies on the learning of the training data so that the training data covers different types of samples as comprehensively as possible, which should enhance the generalization ability of the model. (3) The average detection accuracy of all models on the 2019 sample set is about 80%, which is about 20% lower compared to the accuracy on the 2017 and 2018 test sets, indicating that the detection models are not sufficient to cope with the evolution of samples (including malicious and benign samples), such as malicious feature enhancement and adversarial detection. Therefore, the postmaintenance of the model is important, and the training set should be updated regularly; on the other hand, more robust detection methods are to be further investigated.

**Figure 8:**
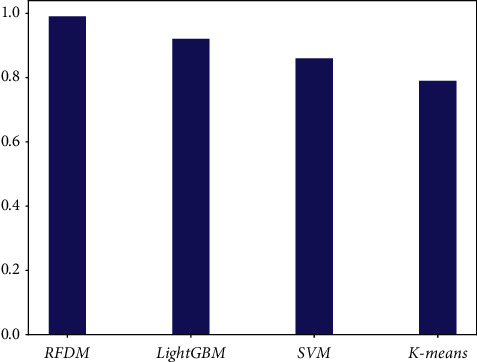
Accuracy comparison of different algorithms.

The proposed method, LightGBM, SVM, and K-means were used experimentally on the Ember 2017 datasets, and a comparison of the accuracy of the various algorithm models is shown in Figure 8. The experimental results also show that the proposed method performs best in Accuracy metrics.

### 4.4. Distributed System Performance Verification

To verify the performance of the improved RFMD parallel algorithm, it was compared with the conventional RFMD algorithm running in a common stand-alone environment. The clustering results of the two algorithms are shown in Table 3. From Table 3, it can be seen that the number of samples correctly classified by the parallel algorithm is larger than that of the traditional algorithm when different classes of Iris in the dataset are classified by the two algorithms; in the clustering of Ember 2017, the detection results of the two algorithms are 95.6% and 99.1%; in the clustering of Ember 2018, the traditional algorithm detects 91.4%, and the parallel algorithm detects 98.6% in accuracy; in the 2019's database, the traditional accuracy is 88.6%, and the parallel algorithm accuracy is 97.1%. The above results show that, compared with the traditional algorithm, the algorithm designed in this paper based on the distributed framework implementation has higher accuracy and better overall detection effect.

## 5. Conclusion

With the increasing growth and spread of malware, the traditional single-model approach no longer meets the needs of malware detection in large-scale network environments. For this purpose, this paper proposes a novel framework for malware detection by machine learning guided by distributed techniques, which overcomes the problem that traditional methods cannot adapt to large-scale networks. The proposed approach constructs a distributed framework that contains a data source layer, a data storage layer, and a data processing layer. The data source layer and data storage layer collect and store traffic logs, threat logs, and PE files, which are the basic work for detecting malware. In the data processing layer, we utilize Hive and Spark for offline computation and real-time analysis, respectively, while the proposed random forest-based malware detection algorithm for anomaly detection is implemented in a timely and effective manner. Experimental results on datasets Ember 2017 and Ember 2017 show that our proposed random forest-based detection method outperforms LightGBM, SVM, and K-means. In distributed performance validation tests, our method significantly outperforms traditional single-model methods, and the real-time performance and accuracy of detection are effectively improved. The method provides technical support for large-scale malware detection on Web platforms. In the future, we plan to investigate deep learning-based malware detection and highly extendable distributed architectures.

## Figures and Tables

**Table 1:** Ember datasets statistics.

Datasets	Quantity (10,000)		
	Malignant samples	Benign samples	Unknown sample
Ember 2017	40	40	30
Ember 2018	40	40	20

**Table 2:** Experimental environment.

Name	Version number or configuration information
Python	3.8
Java	1.8.0
Scala	2.12.10
Spark	3.0.1
Hadoop	3.3.0
Operating system	Windows 10
Number of cluster nodes	1 master node, 3 slave nodes
Node configuration	8 GB RAM, 4-core processor

**Table 3:** Comparison of detection results.

	Number of samples (10,000)	Single model (%)	Distributed model (%)
Ember 2017	40	95.6	99.1
Ember 2018	40	91.4	98.6
Datasets	40	88.6	97.1

## Data Availability

The datasets used during the current study are available from the corresponding author upon reasonable request.
